# Green development of China’s Pan-Pearl River Delta mega-urban agglomeration

**DOI:** 10.1038/s41598-021-95312-z

**Published:** 2021-08-03

**Authors:** Tao Liu, Yue Li

**Affiliations:** 1 School of Economics and Management, China University of Geosciences, Wuhan, 430074 China; 2 China Urban and Rural Holding Group Co., LTD., Beijing, 100020 China

**Keywords:** Sustainability, Environmental economics

## Abstract

Mega-urban agglomerations in developing countries have been main parts of economic development. But at the same time, they have become the most prominent and sensitive areas of resource and environment problems. It is important to clarify the mechanism and driving factors of green growth in mega-urban agglomerations. Based on the panel data of 28 major cities in China's Pan-Pearl River Delta urban agglomeration from 2006 to 2015, this paper evaluates the level of green development of urban agglomeration by green total factor productivity index (GTFP) based on Global Malmquist DEA model, and decomposes GTFP into technological progress, pure technical efficiency change, scale efficiency change and technological scale change. On this basis, this paper constructs a panel econometric model to analyze the influencing factors of GTFP and its decomposition factors. The results show that GTFP of Pan-Pearl River Delta urban agglomeration is growing, and the scale effect caused by technological progress is the main driving factor. Green development in the Pearl River Delta urban agglomeration takes into account efficiency and regional fairness, which causes differences in GTFP growth patterns of sub-urban agglomerations within mega-urban agglomerations. The technological progress and technical efficiency improvement are becoming the main driving force of GTFP growth in relatively backward areas. Furthermore, according to the influencing factors of GTFP and its decomposition factors, mega-urban agglomeration should eliminate internal administrative barriers to build an integrated market. It should also increase the proportion of technology industries in core cities, and give full play to the role of technology spillover effect on surrounding cities. In addition, improving the efficiency of resource and energy utilization is also helpful to promote the transformation of urban agglomeration development from factor-driven to efficiency-driven and innovation-driven. Our research results have implications for the coordinated development of economy and environment in developing countries.

## Introduction

Solow Growth Model believes that economic growth triggered by factor input rather than efficiency improvement is difficult to maintain in the long term^1^. China’s economy has shifted from a stage of rapid growth to a stage of high-quality development, and is now in a critical period of transforming development mode, optimizing economic structure, and switching drivers of economic growth. Facing multiple challenges such as economic growth slowdown, structural adjustment retardation, serious environmental problems, how to realize the transformation of China’s economic development from factor-driven to efficiency-driven and innovation-driven is a major problem at present^2,3^. As the main driving force of China's economic growth, urban agglomerations are not only the areas with high concentration of production factors and the most intense economic activities, but also the most prominent and sensitive areas of resource and environmental problems^4^. Therefore, seeking a way to balance the economic growth and environmental sustainability of urban agglomerations has a powerful meaning for China to promote the transformation of economic development mode under the new economic normal and ecological civilization^5–7^. At the same time, it has important reference significance and demonstration effect for other developing countries facing similar problems.

Traditional total factor productivity can evaluate the quality and productivity level of national (regional) economic growth, but it does not consider the constraints of resources and environment on economic activities and the negative externalities brought by economic activities to the ecological environment, which may lead to the deviation of productivity evaluation results^8^. Therefore, scholars have proposed the green total factor productivity (GTFP) which incorporates environmental pollution and resource constraints into the total factor productivity evaluation system. The GTFP can more objectively evaluate whether the country (region) economic growth is compatible with the resource environment^9,10^. Since then, a large number of literatures have measured and analyzed the GTFP of different countries and regions^11,12^. Among them, the research about China focus on the provincial-level, mainly using the SBM-DEA model that includes undesired output to measure the GTFP^13,14^. These literatures based on provincial data ignore the huge differences of factor endowments and industrial structure between regions, which leads to the lack of realistic basis for the calculation results of GTFP^15^. Therefore, scholars began to pay attention to the measurement of green total factor productivity at the regional level and the causes of differences. Most studies divided China into the eastern, central, and western regions^16,17^, which is reasonable in the context of China's spatial gradient development model. But the emergence of urban agglomerations has broken the provinces’ borders, they reshape the spatial unit of economic activities. So it is necessary to explore the driving force of GTFP from the perspective of urban agglomerations.

The existing literature about the GTFP of urban agglomerations focus on the Yangtze River Delta, the Pearl River Delta, the Beijing-Tianjin-Hebei urban agglomeration and the Yangtze River Economic Belt^18–20^. For example, Yi et al.^18^ used the DEA-Malmquist index method to measure the GTFP of 11 provinces in the Yangtze River Economic Belt from 2004 to 2015, and found that GTFP showed a downward trend in time, and the spatial relevance of GTFP was gradually increasing. Li et al.^19^ used the SBM-DEA model to measure the GTFP of the three major urban agglomerations (Beijing-Tianjin-Hebei, Yangtze River Delta and the Pearl River Delta). The GTFP of Pearl River Delta is higher than the other two urban agglomerations, and resource and environmental constraints are the main reason for the gap in GTFP of three urban agglomerations. It can be seen that most literatures still pay attention to the green development of small urban agglomerations, while few literatures pay attention to the mega-urban agglomerations in China, whose structural complexity and internal differences are far greater than small urban agglomerations. Therefore, it is a frontier research field to study how to achieve coordinated development of economy and environment under the complex characteristics of mega-urban agglomerations^4^.

In 2014, the “Pan-Pearl River Delta Regional Cooperation Declaration (2015–2025)” has been signed, the "Pan-Pearl River Delta Urban Agglomeration" formed. This urban agglomeration occupies 1/5 of the country’s area, over 1/3 of the population and more than 1/3 of the country's GDP (excluding Hong Kong and Macau), which is committed to building the "9 + 2" (Guangdong, Fujian, Jiangxi, and other 9 provinces (regions) and Hong Kong & Macau 2 special administrative regions) cooperative development. Structural optimization, regional cooperation and resource allocation of the Pan-Pearl River Delta urban agglomeration will also have a significant impact on China’s green high-quality economic growth^21^. However, there are significant differences in the economic foundations and resource endowments of the cities. Under the pressure of economic development and environmental constraints, the regional cooperation mechanism within the Pan-Pearl River Delta urban agglomeration is difficult to form, which prevents the Pan-Pearl River Delta urban agglomeration achieving economic green, high-quality, and integrated growth^22^. How to improve the green total factor productivity of the Pan-Pearl River Delta urban agglomeration, and coordinate the resource allocation and industrial development relationship among the inner cities (sub-urban agglomerations)? It’s the key question of green development in mega-urban agglomerations. Therefore, this paper uses the GML (Global Malmquist-Luenberger) index model to measure the GTFP of 28 major cities in the Pan-Pearl River Delta urban agglomeration, and decomposes the main driving forces of the GTFP of each city (sub-urban agglomerations). Then, starting from factors such as factor endowments, industrial structure, etc., we also analyze the causes of different changes in the characteristics of the GTFP of the Pan-Pearl River Delta urban agglomeration; Finally, we put forward some scientific suggestions to promote the green development of the Pan-Pearl River Delta urban agglomeration.

## Methods

### The measurement and decomposition of GTFP

The Malmquist-Luenberger index proposed by Chung et al. ^23^ improved the distance function based on the traditional Malmquist index. It can be used to deal with undesired output^23^. Geometric mean between two periods are usually used when measuring the ML index, which may be no solution to linear programming. Oh^24^ applied the Global Malmquist to ML index and constructed the Global Malmquist-Luenberger index (GML), which effectively avoids the problem of no solution. With reference to Fare et al.^25^ and Ray et al.^26^ on the decomposition method of total factor productivity, under the assumption of constant return to scale, the green total factor productivity change (GTFP) could be calculated and decomposed as follows:

1 $$GTFP_{r}^{t,t + 1} = \frac{{\overrightarrow {S}_{C}^{G} \left( {x^{t} ,y^{t} ,b^{t} ;y^{t} , - b^{t} } \right)}}{{\overrightarrow {S}_{C}^{G} \left( {x^{t + 1} ,y^{t + 1} ,b^{t + 1} ;y^{t + 1} , - b^{t + 1} } \right)}} = GPTC_{t}^{t + 1} \times GPEC_{t}^{t + 1} \times GSTC_{t}^{t + 1} \times GSEC_{t}^{t + 1}$$

Among them, $GTFP_{t}^{t + 1} ,GPTC_{t}^{t + 1} ,GPEC_{t}^{t + 1} ,GSTC_{t}^{t + 1} ,GSEC_{t}^{t + 1}$ represents the change in total factor productivity of the green economy, technological progress, pure technical efficiency change, scale efficiency change, and technological scale change. The specific forms are as follows:

2 $$\begin{aligned} GPTC_{t}^{t + 1} & = \frac{{{{\overrightarrow {S}_{V}^{G} \left( {x^{t} ,y^{t} ,b^{t} ;y^{t} , - b^{t} } \right)} \mathord{\left/ {\vphantom {{\overrightarrow {S}_{V}^{G} \left( {x^{t} ,y^{t} ,b^{t} ;y^{t} , - b^{t} } \right)} {\overrightarrow {S}_{V}^{t} \left( {x^{t} ,y^{t} ,b^{t} ;y^{t} , - b^{t} } \right)}}} \right. \kern-\nulldelimiterspace} {\overrightarrow {S}_{V}^{t} \left( {x^{t} ,y^{t} ,b^{t} ;y^{t} , - b^{t} } \right)}}}}{{{{\overrightarrow {S}_{V}^{G} \left( {x^{t + 1} ,y^{t + 1} ,b^{t + 1} ;y^{t + 1} , - b^{t + 1} } \right)} \mathord{\left/ {\vphantom {{\overrightarrow {S}_{V}^{G} \left( {x^{t + 1} ,y^{t + 1} ,b^{t + 1} ;y^{t + 1} , - b^{t + 1} } \right)} {\overrightarrow {S}_{V}^{t + 1} \left( {x^{t + 1} ,y^{t + 1} ,b^{t + 1} ;y^{t + 1} , - b^{t + 1} } \right)}}} \right. \kern-\nulldelimiterspace} {\overrightarrow {S}_{V}^{t + 1} \left( {x^{t + 1} ,y^{t + 1} ,b^{t + 1} ;y^{t + 1} , - b^{t + 1} } \right)}}}} \\ & \quad \times \frac{{{{\overrightarrow {S}_{C}^{G} \left( {x^{t} ,y^{t} ,b^{t} ;y^{t} , - b^{t} } \right)} \mathord{\left/ {\vphantom {{\overrightarrow {S}_{C}^{G} \left( {x^{t} ,y^{t} ,b^{t} ;y^{t} , - b^{t} } \right)} {\overrightarrow {S}_{V}^{G} \left( {x^{t} ,y^{t} ,b^{t} ;y^{t} , - b^{t} } \right)}}} \right. \kern-\nulldelimiterspace} {\overrightarrow {S}_{V}^{G} \left( {x^{t} ,y^{t} ,b^{t} ;y^{t} , - b^{t} } \right)}}}}{{{{\overrightarrow {S}_{C}^{t} \left( {x^{t} ,y^{t} ,b^{t} ;y^{t} , - b^{t} } \right)} \mathord{\left/ {\vphantom {{\overrightarrow {S}_{C}^{t} \left( {x^{t} ,y^{t} ,b^{t} ;y^{t} , - b^{t} } \right)} {\overrightarrow {S}_{V}^{t} \left( {x^{t} ,y^{t} ,b^{t} ;y^{t} , - b^{t} } \right)}}} \right. \kern-\nulldelimiterspace} {\overrightarrow {S}_{V}^{t} \left( {x^{t} ,y^{t} ,b^{t} ;y^{t} , - b^{t} } \right)}}}} \\ \end{aligned}$$

3 $$GPEC_{t}^{t + 1} = \frac{{\overrightarrow {S}_{V}^{t} \left( {x^{t} ,y^{t} ,b^{t} ;y^{t} , - b^{t} } \right)}}{{\overrightarrow {S}_{V}^{t + 1} \left( {x^{t + 1} ,y^{t + 1} ,b^{t + 1} ;y^{t + 1} , - b^{t + 1} } \right)}}$$

4 $$GSTC_{t}^{t + 1} = \frac{{{{\overrightarrow {S}_{C}^{t + 1} \left( {x^{t + 1} ,y^{t + 1} ,b^{t + 1} ;y^{t + 1} , - b^{t + 1} } \right)} \mathord{\left/ {\vphantom {{\overrightarrow {S}_{C}^{t + 1} \left( {x^{t + 1} ,y^{t + 1} ,b^{t + 1} ;y^{t + 1} , - b^{t + 1} } \right)} {\overrightarrow {S}_{V}^{t + 1} \left( {x^{t + 1} ,y^{t + 1} ,b^{t + 1} ;y^{t + 1} , - b^{t + 1} } \right)}}} \right. \kern-\nulldelimiterspace} {\overrightarrow {S}_{V}^{t + 1} \left( {x^{t + 1} ,y^{t + 1} ,b^{t + 1} ;y^{t + 1} , - b^{t + 1} } \right)}}}}{{{{\overrightarrow {S}_{C}^{G} \left( {x^{t + 1} ,y^{t + 1} ,b^{t + 1} ;y^{t + 1} , - b^{t + 1} } \right)} \mathord{\left/ {\vphantom {{\overrightarrow {S}_{C}^{G} \left( {x^{t + 1} ,y^{t + 1} ,b^{t + 1} ;y^{t + 1} , - b^{t + 1} } \right)} {\overrightarrow {S}_{V}^{G} \left( {x^{t + 1} ,y^{t + 1} ,b^{t + 1} ;y^{t + 1} , - b^{t + 1} } \right)}}} \right. \kern-\nulldelimiterspace} {\overrightarrow {S}_{V}^{G} \left( {x^{t + 1} ,y^{t + 1} ,b^{t + 1} ;y^{t + 1} , - b^{t + 1} } \right)}}}}$$

5 $$GSEC_{t}^{t + 1} = \frac{{{{\overrightarrow {S}_{C}^{t} \left( {x^{t} ,y^{t} ,b^{t} ;y^{t} , - b^{t} } \right)} \mathord{\left/ {\vphantom {{\overrightarrow {S}_{C}^{t} \left( {x^{t} ,y^{t} ,b^{t} ;y^{t} , - b^{t} } \right)} {\overrightarrow {S}_{V}^{t} \left( {x^{t} ,y^{t} ,b^{t} ;y^{t} , - b^{t} } \right)}}} \right. \kern-\nulldelimiterspace} {\overrightarrow {S}_{V}^{t} \left( {x^{t} ,y^{t} ,b^{t} ;y^{t} , - b^{t} } \right)}}}}{{{{\overrightarrow {S}_{C}^{t + 1} \left( {x^{t + 1} ,y^{t + 1} ,b^{t + 1} ;y^{t + 1} , - b^{t + 1} } \right)} \mathord{\left/ {\vphantom {{\overrightarrow {S}_{C}^{t + 1} \left( {x^{t + 1} ,y^{t + 1} ,b^{t + 1} ;y^{t + 1} , - b^{t + 1} } \right)} {\overrightarrow {S}_{V}^{t + 1} \left( {x^{t + 1} ,y^{t + 1} ,b^{t + 1} ;y^{t + 1} , - b^{t + 1} } \right)}}} \right. \kern-\nulldelimiterspace} {\overrightarrow {S}_{V}^{t + 1} \left( {x^{t + 1} ,y^{t + 1} ,b^{t + 1} ;y^{t + 1} , - b^{t + 1} } \right)}}}}$$

Among them, $GPTC_{t}^{t + 1}$ is a change in technological progress, and its value greater than 1 means that compared with the previous production technology, the current production technology is closer to the frontier of global production technology, and technological progress has occurred; $GPEC_{t}^{t + 1}$ is a pure technical efficiency change, and its value is greater than 1. It means that the production efficiency has improved compared with the previous period; $GSTC_{t}^{t + 1}$ is the technological scale change. When the technological scale change is greater than 1, it means the scale effect caused by the change in the disposability of the factor (also can be understood as technological progress)^27,28^; $GSEC_{t}^{t + 1}$ is the change in scale efficiency, and its value greater than 1 indicates that the change in the factor allocation structure (production efficiency) triggers economies of scale. $x^{t}$, $y^{t}$ and $b^{t}$ represent inputs, desirable outputs and undesirable outputs, respectively, $- b^{t}$ indicates the undesirable outputs are converted into expected output by taking negative values. $S_{C}^{G} ,S_{V}^{G}$ are the distance between the input–output combination and the global DEA frontier, subscripts $C$ and $V$ mean constant return on scale (CRS) and variable return on scale (VRS), respectively. When the return to scale is constant, the efficiency change value includes the scale effect. The scale effect of technological progress (frontier movement) and the scale effect of technical efficiency changes (the difference between the $t$ and $t + 1$ technical efficiency of a DMU) are separated by ${{S_{C}^{t} } \mathord{\left/ {\vphantom {{S_{C}^{t} } {S_{V}^{t} }}} \right. \kern-\nulldelimiterspace} {S_{V}^{t} }}$^29^.

### Description of input–output variables

For the definition of the Pan-Pearl River Delta urban agglomeration, previous studies generally used the capital cities of the 9 provinces included in "9 + 2" and the special administrative regions of Hong Kong and Macau as the research objects^30^. However, with the rapid economic development of the Pan-Pearl River Delta urban agglomeration, the inter-regional linkage has become more and more obvious. Many satellite cities have played an important role in the transition and undertaking of regional economic development. This shows that it is not enough to focus on the provincial capital cities. This paper finally determines 28 cities as the objects, and divides them into 6 sub-urban agglomerations according to geographical location, which are shown in Table 1.

**Table 1:** Division of sub-urban agglomeration and corresponding cities.

Sub-urban agglomeration	Cities					
Pearl River Delta urban agglomeration	Guangzhou	Shenzhen	Zhuhai	Dongguan	Jiangmen	Foshan
	Zhongshan	Zhaoqing	Huizhou			
Chang-zhu-tan urban agglomeration	Changsha	Zhuzhou	Xiangtan			
Chengdu-Chongqing urban agglomeration	Chengdu	Chongqing				
Coastal urban agglomeration	Fuzhou	Xiamen	Quanzhou	Zhangzhou	Putian	Ningde
Central Yunnan urban agglomeration	Kunming	Qujing	Yuxi	Chuxiong		
Beibu Gulf urban agglomeration	Nanning	Zhanjiang	Haikou	Beihai		

Coastal urban agglomeration indicates coastal urban agglomeration in the Western Taiwan Strait Region. Due to missing data, Hong Kong and Macau are not included in the scope of this study.

In order to reflect the close relationship between resources, environment and economic activities of the Pan-Pearl River Delta urban agglomeration, this paper adds the related indicators of resources and environment such as land, energy and resources to the input elements, and the undesired output of pollutant emissions is added to the output elements. The input–output variables and description are as follows:

1. *Capital input factors: fixed investment of whole society (100 million yuan)* Capital and labor are the most basic production factors in the production function, and they are also the basis for economic operation. Regarding the calculation of capital stock of cities, Jin et al. (2013) pointed out that the calculation of capital stock of cities under the existing statistical system has problems in unifying key variables. At present, the most significant investment index for cities’ captial stock is still the fixed investment of whole society^31^. Therefore, we uses fixed investment as the index of capital element input. 2. *Labor input factors: the number of employees (10,000 people)* The Pan-Pearl River Delta urban agglomeration has always accounted for most of the foreign investment of China by its advantages in opening market. As a result, a large number of laborers have been attracted to form an industrial structure dominated by labor-intensive industries. Therefore, labor input factors are an important force driving the economic growth of the Pan-Pearl River Delta. We uses the employment population as an indicator to measure labor input^32^. 3. *Land input factors: the area of urban construction land (square kilometers)* land is the carrier for the normal operation of economic activities, and as a resource element endowment, rational use of land is an important link to achieve high-quality urban development. The region of Pan-Pearl River Delta is vast, and the development stages of provinces are quite different. The land resources in Guangdong, Fujian and other provinces are very scarce, and the available urban construction land indicators in the future will be limited, which restricts the urban economic development. With reference to Fang et al.^33^ processing method, this paper selects the urban construction land area as the land element input index^33^. 4. *Energy input factors: total energy consumption (10,000 tons of standard coal)* A typical feature of China’s economy is "high energy consumption". The realization of low energy consumption production is one of the key goals of the green development of urban agglomerations. So the energy cost of economic development should be considered^16^. In view of the fact that cities in China used energy consumption per unit GDP as an indicator before 2010, we calculates the GDP based on 2005 and calculates the total energy consumption of each city. 5. *Resource input factors: total water supply (100 million cubic meters)* Water supply can effectively reflect the degree of resource dependence of cities, so this paper uses the total water supply of each city into the input factor indicators.

1. *Expected output factor GDP* In previous studies, GDP is always used as the expected output indicator to measure the economic benefits of regional production activities. This article takes the regional gross product (GDP) as the expected output, and uses 2005 as the base period for conversion to eliminate the influence of inflation and other factors^13,14,33^. 2. *Undesired output factors: industrial waste water emissions (100 million tons), industrial waste gas emissions (100 million standard cubic meters)* In the GTFP evaluation research, the industrial three types of wastes emissions are generally used as undesired output indicators^34^. Since most municipal solid waste emissions in the Pan-Pearl River Delta region are basically zero, we didn’t add it to the undesired output indicators. This paper chooses industrial wastewater discharge and industrial waste gas discharge as undesired output indicators.

The above-mentioned data comes from the "China Statistical Yearbook", "China City Database", "China Regional Economic Database" and statistical yearbooks of those provinces and cities.

## Results

### GTFP of Pan-Pearl river delta urban agglomeration

**Figure 1:**
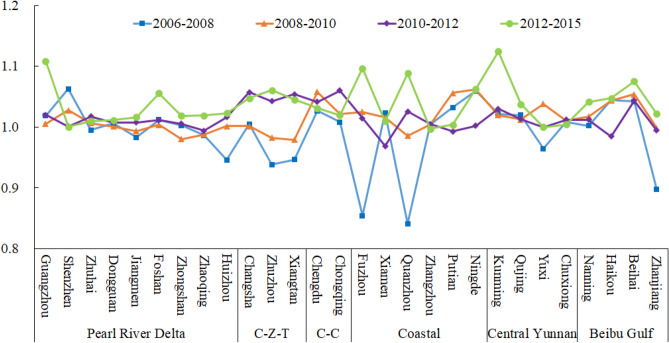
Changes in GTFP of the Pan-Pearl river delta urban agglomeration. *Notes*: C-Z-T means Chang-zhu-tan urban agglomeration. C–C means Chengdu-Chongqing urban agglomeration.

Based on the input–output indicators above, we used the GTFP index model to measure the change of GTFP from 2006 to 2015 in the Pan-Pearl River Delta urban agglomeration. The result is shown in Fig. 1.

On the whole, the geometric mean of the GTFP of the Pan-Pearl River Delta urban agglomeration from 2006 to 2015 was 1.0176, indicating that the GTFP of the Pan-Pearl River Delta urban agglomeration is showing a growth trend, and the growth rate continued to increase (the average GTFP value of each city from 2007 to 2008 is 1.0088, the mean value of GTFP from 2014 to 2015 was 1.0529). In terms of different cities, Kunming, Beihai, Guangzhou, Chengdu, Haikou, Changsha and other cities have average GTFP growth beyond average, indicating that these cities are driving the green growth of the Pan-Pearl River Delta urban agglomeration; The average annual growth of GTFP in cities such as Xiamen Zhuzhou, Xiangtan, Fuzhou and Zhuhai, is greater than 1 but below the average, indicating that the green total factor productivity of these cities is increasing, but it is slightly weaker than the high-growth regions, and cannot effectively drive the green development of the Pan-Pearl River Delta urban agglomeration. The GTFP change of Huizhou, Zhaoqing, Quanzhou, and Zhanjiang is less than 1,which showing non-DEA effective, indicating that the green total factor productivity of GTFP in these four cities is deteriorating, which is not conducive to the green growth of the Pan-Pearl River Delta urban agglomeration.

From the perspective of sub-urban agglomerations, due to the large differences in samples contained of sub-urban agglomeration, we selected Pearl River Delta urban agglomeration, Coastal urban agglomeration, Central Yunnan urban agglomeration and Beibu Gulf urban agglomeration with relatively rich urban samples to analyze the unbalanced characteristics of internal GTFP: the variance of GTFP within Pearl River Delta urban agglomerations is small, and the GTFP of most cities are increasing, which shows that the green development of Pearl River Delta urban agglomerations takes into account efficiency and fairness; The GTFP of Coastal urban agglomeration are converging, and the variance decreases gradually, indicating that the Coastal urban agglomeration is changing from polarized green development to balanced development. The variance of GTFP in Central Yunnan urban agglomeration is increasing sharply, which shows a polarized green development pattern is being formed with Kunming as the center. However, the GTFP of other cities in Central Yunnan urban agglomeration are also close to 1, indicating that the catch-up effect between cities exists significantly; The divergence of GTFP in Beibu Gulf urban agglomeration is the most serious, basically showing a polarized development pattern. With the policy support of Beihai and Haikou, GTFP is rising rapidly, while Zhanjiang, as the main destination of industrial transfer in the east, is seriously backward, which shows that developing countries are bound to have relatively backward cities to undertake high-pollution industries due to industrialization needs. It’s also a trade-off made by developing countries under the pressure of carbon emission reduction.

### Decomposition of GTFP

Most cities in the Pan-Pearl River Delta urban agglomeration show a trend of green economic growth. From the decomposition of the GTFP index, it can be seen that the growth of green total factor productivity comes from both the scale effect brought by the expansion of production activities and the production efficiency brought about by technological progress and the improvement of development quality. Therefore, it is impossible to judge the driving force of the economic green growth of the Pan-Pearl River Delta urban agglomeration from the changes in the GTFP index alone. The total factor productivity of the green economy needs to be decomposed in the form of formulas 2–5, the results show that the average values of GPTC, GPEC, GSTC and GSEC are 1.0085, 1.0041, 1.0185 and 0.9988 respectively. It can be seen that the main factors driving the growth of GTFP in the Pan-Pearl River Delta urban agglomeration are triggered by scale of technological progress. Economies of scale indicate that the emergence of new technologies within the Pan-Pearl River Delta urban agglomeration can generate positive externalities for the green growth of the entire regional economy, which benefits from the cooperation and sharing mechanism of new technologies in various regions. From the two decomposition items of technological progress, it can be seen that technological progress and pure technical efficiency are both greater than 1, indicating that the Pan-Pearl River Delta urban agglomeration has shown technological progress as a whole, and the factor utilization efficiency has also increased significantly, which has played an important role to the growth of GTFP in the Pan-Pearl River Delta urban agglomeration. Among the four decomposition items, only GSEC is less than 1, indicating that the scale efficiency of the Pan-Pearl River Delta urban agglomeration has deteriorated at this stage. Although the pure technical efficiency has improved, it has not produced a scale effect. This may be due to the insufficient opening of the production factor market caused by insufficient factor mobility.

**Figure 2:**
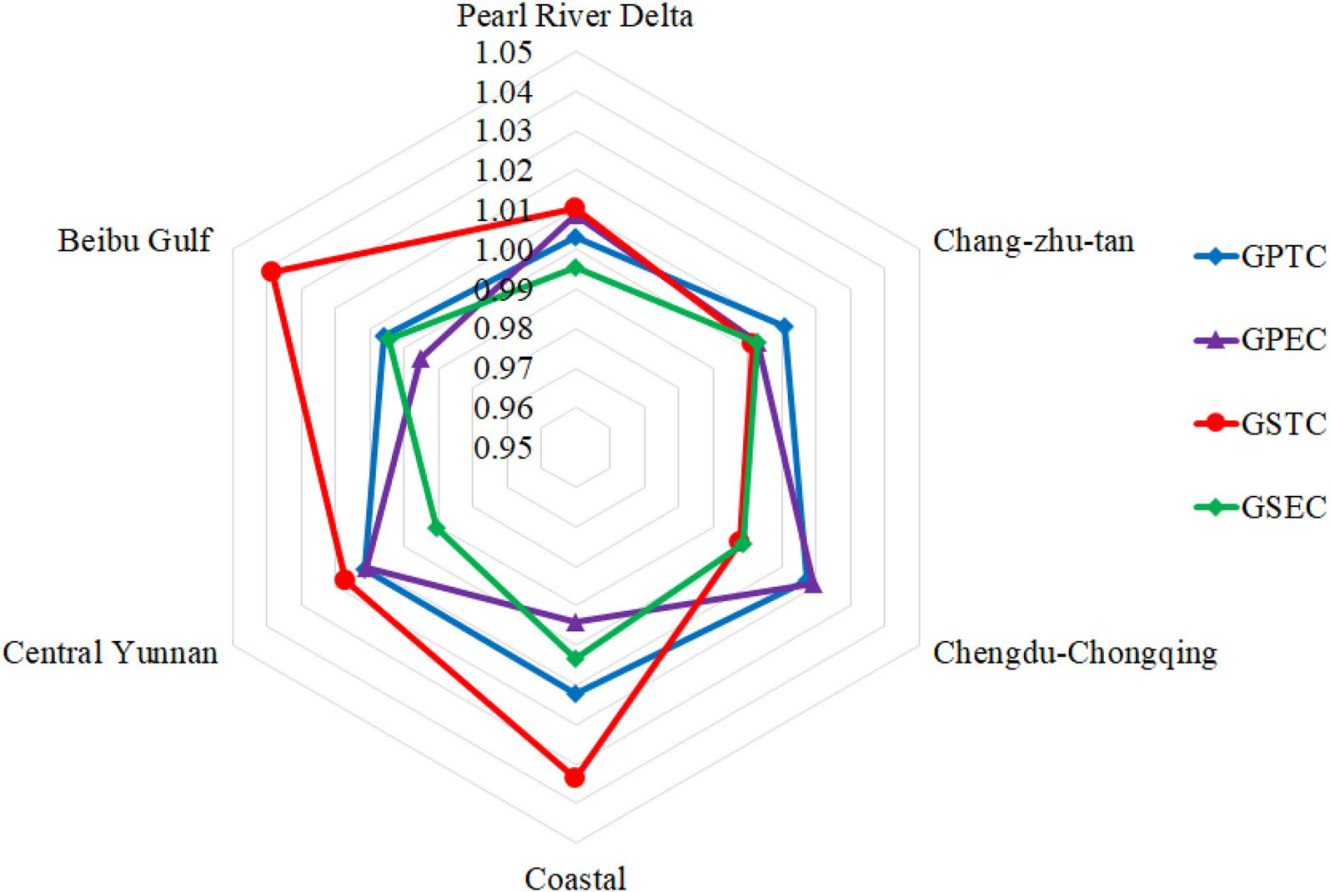
Decomposition of green total factor productivity changes in sub-city groups (2006–2015 average).

From Fig. 2, we can see the relative contribution of each sub-city group to the different decomposition items of the GTFP of the Pan-Pearl River Delta urban agglomeration. Among them, the GPTC of each sub-city group is greater than 1, which is represented by technological progress. Chengdu-Chongqing urban agglomeration and urban agglomeration on the west side of the Straitsare are the most prominent, and the Pearl River Delta urban agglomeration is at the bottom. This shows that the traditional industrial urban agglomeration accelerateing technological learning and industrial upgrading and transformation is the main driving force for the technological progress of the Pan-Pearl River Delta urban agglomeration; From the perspective of GPEC, the growth rate of technical efficiency of the Chengdu-Chongqing urban agglomeration ranks first, followed by the central Yunnan urban agglomeration and the Pearl River Delta urban agglomeration, but the urban agglomeration on the west coast of the Straits and the Beibu Gulf urban agglomeration have shown pure technical efficiency deterioration. Among the GSTC of the sub-cities, only the Chengdu-Chongqing urban agglomerations have experienced scale-tech regressions, indicating that the technological progress within the Chengdu-Chongqing urban agglomerations has not produced scale effects, and the technological level is moving towards constant returns to scale. It is necessary to further strengthen the regional sharing mechanism of technological progress and construct to achieve economies of scale through the positive externalities of technology spillover to economic growth. In addition, the Chengdu-Chongqing region, as a transitional area between the Pan-Pearl River Delta urban agglomeration and the northwestern region, plays an important pivotal role in strengthening the cooperative development of the northwest and southeast regions. Therefore, the Chengdu-Chongqing region urgently needs to open up technology sharing channels between cities and introduce the advanced technology from the southeast into the northwest, and then the megacities have a radiating effect on the high-quality growth of China’s economy. In the GSEC, the scale and efficiency of the Changsha-Zhutan, the city groups on the west coast of the Taiwan Strait, and the Beibu Gulf city groups show an increasing trend, indicating existing factors of the allocation structure has formed a certain scale effect within the three urban agglomerations, but the pure technical efficiency of the urban agglomerations on the west coast of the Straits and the urban agglomerations outside the northern part of the Straits is showing a downward trend. It is necessary to be wary of the decline in the utilization efficiency of factors that causes the "scale effect" to become "crowding effect"^35^. The scale efficiency of the Pearl River Delta urban agglomeration, the Chengdu-Chongqing urban agglomeration, and the central Yunnan urban agglomeration is less than 1, indicating that the increase in pure technical efficiency has instead appeared diseconomies of scale, which indicates that these urban agglomerations have element redundancy, and the improvement of technical efficiency is not yet complete. To digest the input redundancy, the scale of input elements should be appropriately reduced to give full play to the scale effect of technical efficiency improvement.

## Further analysis of the influencing factors of GTFP

### Influencing factor index selection

The choice of the path to achieve innovation drive depends on the rationality of external conditions such as urban factor endowments and institutional environment^36^. Different exogenous environments of urban agglomeration determine the driving mode of green development of urban agglomeration. These exogenous environments can be summarized into four aspects: factor endowment and factor allocation pattern, market environment, science and education level and government environmental regulation. Among them, factor endowment and factor allocation pattern are the factor sources of economic operation of urban agglomeration, which shape the utilization mode of various production factors of urban agglomeration and also determine whether urban agglomeration prefers intensive development or extensive development; The meaning of market environment is the marketization level and opening degree of urban agglomerations, which determines whether urban agglomerations tend to develop in an open or closed way; The science and education level can be reflected by the overall educational and scientific research level of urban agglomeration, symbolizing the quality of human capital of urban agglomeration, and determining whether the development of urban agglomeration tends to be high-tech or low-tech; The government environmental regulation is more reflected in the government's investment in environmental governance in green development, which determines whether the development of urban agglomeration pays attention to energy conservation and emission reduction. Specific indicators are selected as follows:

1. *Factor endowment and factor allocation pattern* Regional factor endowments include capital factor endowments and natural resource factor endowments^37^. This article uses industrial structure to reflect the differences in capital factor allocation, and uses energy consumption intensity, land use intensity and water resources endowments as natural resource factor endowments. The proxy variable of: Capital factor allocation method (industrial structure): The capital factor allocation method not only determines the regional industrial structure, but also plays an important guiding role in the future development of the region. Because the allocation of capital factors is difficult to measure, this article refers to the research of Qian et al.^13^ and others, and uses the proportion of the secondary industry (SIR) and the proportion of the tertiary industry (TIR) to measure the bias of the allocation of capital factors of the city; Resources and energy Consumption Intensity (Utilization of Resources and Environment): In order to fully reflect the differences in resource endowments and environmental characteristics of each city, this paper selects energy consumption intensity (TECG), land use intensity (EUII) and water resource use intensity (PWCW) to measure how much degree are regional economic activities dependent on the resource environment.
2. *Market environment (FDI)* Market openness helps to accelerate the flow of factors within and between urban agglomerations, and is a potential driving force for the coordinated development of regional economies^38^. This article chooses FDI (Foreign Direct Investment) to measure the degree of market openness of different evaluation units.
3. *Science and education level (R&D/EDU)* The level of investment in science and technology and the level of education reflect a region's emphasis on technological progress and labor quality. The level of science and education is closely related to the high-quality development of the city^39^. This article uses R&D investment in GDP to measure the level of science and technology investment, and the proportion of people with a college degree or above in the population to measure the regional education level.
4. *Government environmental regulation (ENVL)* Generally speaking, strengthening environmental regulations is conducive to the transformation of regional production methods to more efficient and green methods, thereby promoting green economic development^40^. In this paper, the percentage of fiscal investment in pollution control in fiscal expenditure in GDP is used as a proxy variable for the intensity of environmental regulations.

The above-mentioned data comes from the corresponding year "China Statistical Yearbook", "China City Database", "China Regional Economic Database" and statistical yearbooks of various provinces and cities. Descriptive statistics are shown in Table 2.

**Table 2:** Explanation of each explanatory variable and its descriptive statistical analysis.

Variable	Code	Unit	Observations	Mean	SD	Max	Min
The proportion of secondary industry	SIR	%	252	0.3783	0.1330	0.7370	0.0260
The proportion of tertiary industry	TIR	%	252	0.5895	0.1510	0.9549	0.2034
Energy consumption intensity	TECG	tce/10^4^ yuan	252	0.0928	0.0458	0.2415	0.0201
Land use intensity	EUII	km^2^/10^9^ yuan	252	13.6566	9.3966	56.6672	1.4791
Water resource utilization intensity	PCWC	m^3^/10^4^ yuan	252	0.6945	0.2649	1.9689	0.1952
Foreign investment level	FDI	%	252	0.4056	0.2954	1.6150	0.0049
Education level	EDU	%	252	0.0644	0.0586	0.2414	0.0043
Research investment level	R&D	%	252	0.0099	0.0106	0.0504	0.0003
Government environmental regulation	ENVL	%	252	0.0077	0.0074	0.0334	0.0001

The value and quantity indicators in the table are all converted into constant prices in 2005.

### Internal factors of Pan-Pearl river delta urban agglomeration’s GTFP

Green total factor productivity change (GTFP), technological progress (GPTC), pure technical efficiency change (GPEC), scale efficiency change (GSEC) and technology scale change (GSTC) are the explained variables, and the above influencing factors are explanatory variables to build a panel regression model. The fixed/random effects model is selected based on the Hausmann test. In the empirical test, all regression models used the robustness standard deviations clustered to the city individual level for regression. Table 3 reports the estimated results of each model.

**Table 3:** Estimation results of factors affecting changes in green total factor productivity.

Variables	Model (1)	Model (2)	Model (3)	Model (4)	Model (5)
	GTFP	GPTC	GPEC	GSTC	GSEC
*SIR*	−0.5598*** (−2.1480)	−0.0476 (−0.1013)	−0.0828 (−0.4371)	0.0587 (0.1731)	0.1208 (0.2640)
*TIR*	0.5371*** (2.0758)	0.2163 (0.4632)	−0.1162 (−0.6296)	0.0087 (0.0263)	0.3103** (0.6798)
*TECG*	−0.0071** (−2.3923)	0.0029 (0.5546)	−0.0002 (−0.1656)	−0.0049* (−0.9225)	−0.0023 (−0.4228)
*EUII*	−0.0007 (−0.5932)	0.0018 (0.8253)	0.0005 (0.6819)	−0.0007 (−0.5581)	0.0017 (0.7391)
*PCWC*	−0.0001 (-0.9846)	0.0001 (0.5712)	−1.73E−06 (−0.0276)	4.90E−05 (0.4387)	−0.0002 (−0.8534)
*FDI*	0.0451** (2.1236)	0.0102 (0.2651)	0.0091 (0.5271)	−0.0131 (−0.4233)	0.0229* (0.9269)
*EDU*	−0.4988 (−1.2936)	0.4957* (0.7121)	−0.0170 (−0.0733)	0.1819 (0.4383)	0.7857** (1.0618)
*R&D*	4.4786** 2.4839)	7.4667*** (2.2939)	1.3040 (1.7683)	−1.7409 (−1.3197)	4.7420** (1.3185)
*ENVL*	−0.0443 (−0.0149)	−8.3739** (−1.5548)	−2.0073 (−1.1931)	1.4023 (0.4659)	−6.2524* (−1.2330)
Observations	252	252	252	252	252
Individual fixation	YES	YES	YES	YES	YES
Fixed time	NO	NO	NO	NO	NO

***, **, *respectively represent the significance level of 1%, 5%, and 10%, and the standard deviation of cluster robustness is in parentheses, and Observations represent the number of samples.

From the perspective of the influencing factors of green total factor productivity (model 1 in Table 3), the proportion coefficient of the secondary industry is significantly negative, and the coefficient of the tertiary industry is significantly positive, indicating that increasing the proportion of investment in the tertiary industry and appropriately reducing the proportion of investment in secondary industry (especially the proportion of investment in high-polluting industries) will help increase the green total factor productivity of the Pan-Pearl River Delta urban agglomerations. The allocation of capital factors in the urban agglomerations should continue to tilt towards the service industry and high-tech industries. The energy consumption intensity coefficient is significantly negative, indicating that urban agglomerations should reduce energy consumption intensity and improve energy utilization efficiency in order to drive the improvement of the extensive production mode of urban agglomerations and promote the green development of urban agglomerations. Both the land use intensity and water use intensity coefficients are not significant, indicating that the land and water use efficiency has basically reached the optimal level under the current productivity, and the marginal contribution to the growth of green total factor productivity is basically zero. Resource elements and the green total factor productivity growth in Pan-Pearl River Delta urban agglomerations are decoupling^41^. The coefficient of the degree of market opening is significantly positive, indicating that expanding market opening is conducive to the improvement of green total factor productivity in the Pan-Pearl River Delta urban agglomeration. In the science and education level, the coefficient of education level is not significantly negative. This may be caused by the mismatch between the increase of high-quality talents and the insufficient talent absorption department. Shortage of jobs and low salaries are important reasons for this phenomenon. The science and technology input coefficient is significantly positive. Increasing R&D input can effectively improve green total factor productivity, indicating that the Pan-Pearl River Delta urban agglomeration has a higher conversion efficiency of science and technology input and output, and has improved the driving effect of science and technology on economic growth. The environmental regulation coefficient is not significantly negative, indicating that at this stage, the intensity of environmental governance in the Pan-Pearl River Delta urban agglomeration has not effectively guided the transformation of economic growth to a green and efficient way. In view of the complex operating mechanism of regional environmental regulation, we will not discuss it here. This article preliminarily believes that the “race to the bottom” of environmental regulation policies under the decentralized system in our country has weakened the effectiveness of environmental governance.

In models (2)–(5), the four decomposition items are also affected by various factors to varying degrees. Judging from the effects of various influencing factors, the coefficient of the tertiary industry in model (2) is not significantly positive, and the coefficient of the tertiary industry proportion in model (5) is significantly positive, indicating that the rising proportion of the tertiary industry not only promotes technology to a certain extent, but progress is more conducive to the scale effect of technological progress. This is not only because of the high-tech industry as an important part of the tertiary industry, which can promote technological progress, but also because the development of the tertiary industry depends on the formation of industrial clusters, which is conducive to optimizing factor allocation and expanding technology spillover effects. The scale effect of the agglomeration of factors can be brought to the best state. In terms of energy consumption intensity, only the coefficient of the corresponding term of model (4) is significant and negative, indicating that excessive energy consumption intensity mainly inhibits the improvement of urban agglomeration scale technical efficiency, which in turn hinders the growth of green total factor productivity. In models (2)–(5), the coefficients of land and water use intensity are not significant and close to 0, indicating that the decoupling of resource element input and green total factor productivity has no effect on the enhancement of the decomposition items of green total factor productivity. The degree of market openness is significantly positively correlated with the efficiency of scale technology, indicating that expanding market opening is an important condition for the effect of scale^27^. The coefficient of education level in model (2) and model (5) is significantly positive, indicating that the improvement of education level is not only conducive to the improvement of pure technical efficiency, but also can exert the scale effect brought by the improvement of pure technical efficiency. This is due to colleges and universities that can significantly improve the technical level of the labor force in the region. At the same time, the school has a strong spatial attraction to the population in the region, which promotes the construction of regional infrastructure and other related fields, and is an important carrier for the effect of scale. R&D investment is significantly positively correlated with GPTC and GSEC, indicating that increasing scientific research investment is still an effective way to promote technological progress, and at the same time, it is conducive to promoting high-efficiency production models in the region and forming a scale effect of pure technical efficiency improvement. There is a significant negative correlation between the intensity of environmental regulation and technological progress. This may be due to the "U" curve relationship between environmental regulation and technological progress in the eastern region^42^. The overall intensity of environmental regulation is generally low (mean value is 0.0077, which is in the first half of the curve of U) which cannot effectively promote technological progress, and therefore cannot promote the growth of green total factor productivity.

## Conclusions and implications

Based on the panel data of 28 major cities in the Pan-Pearl River Delta urban agglomeration from 2006 to 2015, this paper estimates the changes in green total factor productivity and its decomposition items in the Pan-Pearl River Delta urban agglomeration, and uses a panel measurement model to analyze the green total factor productivity and its decomposition items. The main conclusions are as follows:

1. On the whole, the green total factor productivity of Pan-Pearl River Delta urban agglomeration is increasing. The green total factor productivity of the six sub-urban agglomerations has also increased year by year. Among them, the variance of GTFP of the Pearl River Delta urban agglomeration is small, and green development takes into account efficiency and fairness; The GTFP of the urban agglomeration on the west side of the Taiwan Straits converges in time series, and the variance decreases gradually, indicating that the urban agglomeration on the west side of the Taiwan Straits is changing from polarized green development to balanced development. The variance of GTFP in central Yunnan urban agglomeration is increasing year by year, and it is forming a polarized green development model with Kunming as the core. The GTFP of other cities is also close to 1, indicating that the catch-up effect between cities exists significantly; The differentiation of GTFP in Beibu Gulf urban agglomeration is the most serious, which basically presents a polarized development model. This fully reflects the obvious differentiation characteristics within megacities.
2. According to the decomposition results of green total factor productivity in Pan-Pearl River Delta urban agglomeration, it can be found that scale technology change, pure technology change and pure technology efficiency are all greater than 1, among which scale technology change is the most important factor for the growth of green total factor productivity in Pan-Pearl River Delta urban agglomeration, but scale efficiency is deteriorating, which shows that there is redundancy in factor input. The technological progress of the six sub-urban agglomerations is greater than 1. Among the typical urban agglomerations, the technological progress of the west coast urban agglomerations is the strongest, while the technological progress of the Pearl River Delta urban agglomerations is relatively weak, which shows that accelerating technological learning and industrial upgrading and transformation of traditional industrial urban agglomerations is the main driving force for the technological progress of Pan-Pearl River Delta urban agglomerations. The deterioration of pure technical efficiency in urban agglomerations on the west coast of the Taiwan Straits and Beibu Gulf indicates that the existing production technology is difficult to digest excessive factor inputs, and the internal adaptability of the production system is reduced, resulting in low economic efficiency.
3. From the influencing factors of green total factor productivity, the proportion of secondary industry is significantly negatively correlated with green total factor productivity, while the proportion of tertiary industry is significantly positively correlated with green total factor productivity. Cultivating environment-friendly industries is still the most effective way to promote green development. There is a significant negative correlation between energy consumption intensity coefficient and green total factor productivity, which shows that extensive energy consumption will seriously hinder the growth of green total factor productivity in urban agglomerations; The coefficient of land use intensity and water resources utilization intensity are not significant, which may be that most cities are in the late stage of industrialization, and the utilization technology of traditional elements such as land and water resources is relatively mature, which has no significant effect on green development. Expanding market opening is conducive to the improvement of green total factor productivity in Pan-Pearl River Delta urban agglomeration, which is inconsistent with the traditional "pollution paradise" hypothesis^43^. The relationship between market opening degree and environmental pollution in mega-urban agglomeration needs further study; The coefficient of science and technology investment is significantly positive, and increasing R&D investment can effectively improve green total factor productivity, which may be related to enhancing social awareness of environmental protection and the close connection between science and education industry and technological progress.
4. Among the four decomposition items of green total factor productivity, the proportion of tertiary industry is significantly positively correlated with technological progress, indicating that the upgrading of industrial structure is one of the main driving forces of technological progress in urban agglomerations; There is a significant negative correlation between energy consumption intensity and scale technical efficiency, which may be that there are obstacles in the conversion between various energy sources, which leads to low energy utilization efficiency and reduces the potential of scale effect caused by the improvement of technical efficiency; There is a significant positive correlation between market openness and scale technical efficiency, which shows that improving market openness and building an integrated market within urban agglomerations are conducive to technology spillover; The improvement of education level is not only conducive to improving pure technical efficiency, but also brings into play the scale effect brought about by the improvement of pure technical efficiency; Increasing R&D investment can promote technological progress and bring into play the scale effect of pure technical efficiency improvement. There is a significant negative correlation between the intensity of environmental regulation and technological progress, which may be due to the overall low intensity of environmental regulation in the Pan-Pearl River Delta urban agglomeration, which shows a cost compensation effect on the environmental regulation of enterprises and inhibits technological innovation.

Based on the above conclusions, this article has the following recommendations: (1) Within the Pan-Pearl River City urban agglomeration, the administrative barriers within the Chengdu-Chongqing urban agglomeration and between other urban agglomerations need to be further broken, and the Chengdu-Chongqing urban agglomeration should be used to connect the northwest and southeast regions as the role of a bridge for collaborative development; The urban agglomerations on the west side of the Straits and the urban agglomerations outside the northern part of the Straits should actively explore new models for improving the efficiency of factor utilization and avoid the "crowding effect" of input factors. The Pearl River Delta urban agglomeration should appropriately spread the factors of production to the surrounding areas and give full play to the scale effect of factor allocation. (2) The capital elements of the Pan-Pearl River Delta urban agglomeration should be more inclined to the service industry and high-tech industry as the main tertiary industry. In particular, increasing investment in education and R&D will significantly promote technological progress and promote technological efficiency. At the same time, relying on the radiation function of educational institutions and research institutes will have a scale effect on the overall green total factor productivity of the region. (3) The Pan-Pearl River Delta urban agglomeration should continue to expand the degree of market openness, accelerate the flow of factors, and optimize the allocation of factors in the urban agglomeration based on market mechanisms. But at the same time, it is necessary to appropriately raise the investment threshold to minimize the entry of high pollution and high energy consumption foreign businessmen, and the phenomenon of "pollution refuge" appears, which will put pressure on the resources and environment of urban agglomerations. (4) There is a close relationship between energy consumption intensity and green total factor productivity growth, which is obviously not conducive to the economic development of the Pan-Pearl River Delta urban agglomeration and the decoupling of resources and energy. Therefore, the proportion of environmentally friendly industries such as scientific research will be further increased, and the proportion of Pan-Pearl River Delta cities will be reduced. Reducing the dependence of the economic growth of agglomerations on the input factors of resources and energy will effectively promote the transformation of the urban agglomeration development model from factor-driven to efficiency-driven and innovation-driven.
